# Pulp Mummification

**Published:** 1899-08

**Authors:** Theodore Soderberg

**Affiliations:** Dentist, Sydney, Australia.


AMERICAN JOURNAL

—OF—

DENTAL SCIENCE.

VOL. XXXIII. BALTIMORE, AUGUST, 1899. NO. 4.

### Selections.

ARTICLE I.

### PULP MUMMIFICATION.

THEODORE SODERBERG, DENTIST, SYDNEY, AUSTRALIA.

(From *The Dental Cosmos*).*

In the *Dental Cosmos* for May, 1893, Dr. W. E, Christensen communicated some comments on the Witzel and Herbst methods of treating devitalized pulps, and pointed out that the real effect aimed at by the two German dentists was the mummification of the pulps left untouched in root canals

Previous to reading about "this treatment, which we may call simple, and at the same time scientific," I had

*Since publishing an article by Dr. Waas in which he referred to this paper by Dr. Soderberg we have been inundated with requests for information contained therein. Our correspondents have been referred to the publishers of the *Cosmos* with the result that the particular number of the *Cosmos* has been exhausted. As we are still receiving requests for the article, it is now reprinted to satisfy the demand.—EDITOR.

followed the usually thought and practiced methods of treating devitalized pulps, viz., to give my patients just the amount of strain and pain equivalent to the amount of my own bulldog perseverance with that exquisite instrument of torture—the nerve broach; then *trying* to fill the canals with gutta-percha or asbestos fibers saturated with some antiseptic; and then—well, the final result was not always my patient's comfort any more than my own glory.

I discarded the old method and adopted Dr. Witzel's, commencing by using the following modified formula (his paste, as formulated by Dr. Christensen, being too thin to be workable):

| | |
|---|---|
| Hydrag. bichor. corr., gr. xxx; | Ol. Menth. pip., gtt. j; |
| Morph. muriat., gr. xv; | Ol. caryophyl., gtt. j; |
| Ac. phenyl., gr. x; | Alum. exsiccat., q. s., to make stiff paste. |

This paste had, however, the drawback which all pastes containing mercury have—it caused discoloration of the tooth, at least when used in connection with steel instruments and amalgam.

Shortly afterward [September, 1893), the *Dental Cosmos* brought out Dr. W. D. Miller's valuable contribution, "Concerning Various Methods Advocated for Obviating the Necessity of Extracting Devitalized Tooth-Pulps," and I at once commenced to experiment with different pastes to find one which would cause mummification of the pulp without discoloration of the tooth.

The properties of an ideal mummification paste can be shortly described thus:

1.—It must contain an antiseptic sufficiently strong to prevent decomposition taking place while mummification sets in. Once mummified, the pulp is (so at least, I believe) not very likely to become decomposed and putrid.

2.—It must contain an ingredient which will, as quickly as possible, cause mummification (drying, shriveling) of the pulp tissues.

3.—It must contain a substance which, in conjunction with the other ingredients, will impart a white color to the mummified pulp, and prevent discoloration of the tooth.

4.—It must contain an agent capable of binding the whole compound together in a pasty state, and making it penetrate deeply and quickly.

Dr. Miller enumerates yet other points; but the above are, I believe, sufficient for all practical purposes.

Besides Dr. Witzel's paste, as given above, I have experimented with three of Dr. Miller's formulæ, using glycerol as the binding agent, viz:

| | | |
|---|---|---|
| Sublimate } equal parts;<br>Thymol } | Sublimate }<br>Thymol } equal parts;<br>Tannin } | Ol. cassia,<br>Thymol, q. s. |
| Glycerol, q. s. | Glycerol, q. s. | |

Here, again, the advantage is—as pointed out by Dr. Miller—the discoloration of the tooth, bluish-black by the mercury, yellowish-brown by the oil of cassia. My test tubes further show that where tannin is used in connection with either of these, or any other paste—especially if glycerol be used as a binding agent—the discoloration is more marked.

Experiments with twelve other pastes, first in ten tubes, next with freshly extracted teeth, proved to my preliminary satisfaction that the following formula was the most reliable on the four points above enumerated:

| | |
|---|---|
| Glycerol, ʒi; | Zinc oxide, q. s. to make stiff paste. |
| Dried alum, ʒi; | Thymol, ʒi; |

In this paste the thymol acts as the antiseptic, the alum as the mummifying agent, the zinc oxide as the coloring medium, and the glycerol as the binding and penetrating agent.

Bearing in mind Dr. Miller's favorable recommendation of thymol, I adopted it as my antiseptic, and I fancy that I shall not have cause to regret my choice. My reason for adopting dried alum was that its tanning properties are far superior to those of tannin or any other tanning agent—a statement which any practical tanner will indorse.* Further, dried alum does not discolor the paste,

*I have it on the authority of the senior partner of the largest tanning establishment in Sydney, (Ludowici & Sons), that an ox-hide can be tanned with dry alum in less than one-tenth the time that any other substance consumes in the process.

while tannic acid, if substituted for the alum in the above mixture, produces a dark-brown paste.

I need hardly point out that glycerol with its great affinity for moisture and its well known penetrative power, is an excellent carrying agent for all mummifying pastes; further, that no better coloring medium can be found for the purpose in view than zinc oxide.

The entire paste is, finally, non-irritating; I have, in fact, continually used it for other purposes, e. g., in deep cavities between pulp and filling material, etc.

I have used this particular paste for over twelve months, and, *so far*, not one single case out of a total of ninety-seven has given any after trouble (alveolar abscess). And here I may be allowed to state that neither have I, so far, had any after trouble with any of the cases treated with Mr. Witzel's paste (from August, 1893, to date). I am perfectly well aware that neither one year, nor two, is sufficient time to warrant a guarantee of absolute success, and I acknowledge the truth of Dr. Miller's words, "Granted that the pulp becomes sterilized by the operation, this does not say that it remains sterile indefinitely."

I must here point out that my claims as a bacteriologist are *nil*, and as far as experimental chemistry is concerned, my knowledge is only that of the average educated dentist. I am unable to make experiments with mummified pulps on infected agar culture, and I am unable to give the "higher scientific reasons" why and how the paste acts as it does. All I know is that it *does* act mummifying on the dead pulp, and that twelve months after that mummy is still a mummy, and not a soft, sinking horror.

My patients mostly belong to the so-called middle and working classes. For divers reasons, they are not as careful with their teeth as are the members of the more cultured and moneyed classes; they don't as a rule, think of visiting the dentist except when an exposed pulp acts as a vivid reminder. In short, my practice is such a one that

I have to resort to my devitalizing paste very frequently. Hence excellent opportunities to see and observe the effect of the mummification paste. (And hence, also, the "kind o' sickly smile" wherewith I point out to my pupils Dr. Miller's angelic advice: "One or two cases every month — at least for the first year or two—is all that a careful dentist ought to risk in private practice." It is, however, a great consolation to me to read in the same article that over two hundred cases were treated with Dr. Miller's paste at the Dental Institute of the University of Berlin, with only one failure on record). I have tested the action of the mummification in the way that conforms best with my bent of mind and my inferior staadard of scientific education: I have removed test fillings three, six, nine and twelve months after the pulp treatment took place. In all cases the same satisfactory result observed—mummification of the pulp. One case I especially wish to record. I had occasion to extract an upper third molar treated seventeen days previously. On immediately splitting the tooth, I found the pulp in the root canals perfectly mummified down to the very foramina. Whether the experiments are carried out with the teeth *in situ*, or with extracted teeth, the mummified pulps always present the same appearance, viz.; a perfectly dry, parchment-like mass, with a faint odor of thymol, and a whitish color.

My mode of procedure is as follows: After the pulp has been perfectly devitalized (arsenic, cocaine, alum, equal parts, glycerol, q. s., sealing with sticky wax), I open up the main pulp chamber and drill out its dead contents, leaving the root canals untouched. I then fill the chamber with paste, and with a flexible Donaldson bristle gently prick the paste into the pulps left in the canals (this, however, is not absolutely necessary). I now seal with cement and insert amalgam or gold, as the case may be. I use rubber dam or cup where possible, otherwise Sperling's rolls—the main thing to keep out the saliva, at least until the first piece of amalgam has been inserted and burnished round.—*Items of Interest.*

